# Hints and Improvements in the Practice of Medicine and Surgery

**Published:** 1799-08

**Authors:** 


### HINTS AND IMPROVEMENTS
### IN THE PRACTICE OF
### MEDICINE AND SURGERY.

### *To the Editors of the Medical and Physical Journal.*

GENTLEMEN,

I AM happy in having it in my power to communicate another instance of the efficacy of the *nitrat of silver*, in the cure of *epilepsy**; and I make no apology for presenting '*one solitary case*', as I think every particular, however minute, respecting the cure of this formidable disease, is worthy of attention. The following are extracts from more copious notes on the case.

May 21st, 1799, A. W. a woman, thirty-six years of age, of a strong, robust habit, has been for four years affected with fits, which, though at first less frequent, have for some time past been scarcely absent for two days together, and yesterday she had three very severe fits. Her general health is tolerably good, except that her appetite is impaired. As, according to her account, a variety of medicines had been tried without success, I resolved immediately to begin the use of the *nitrat of silver*, of which she was ordered to take half a grain, twice a day, in the form of a pill.

24th. Had a fit yesterday, as severe as usual: feels no uneasiness from the pills.—Continues the pills, but the dose increased to a grain and a half a day.

30th. Has had no fit: no uneasiness from the pills: complains of costiveness.—Continues the pills; and the belly kept open by the use of the common pill.

June 10. Catamenia have this day appeared—says that formerly the fits were more frequent and severe at that period: no return of the fits; appetite much improved.—Continue.

28th. Has had no return of the fits. Has this day returned to the country, to all appearance perfectly cured; but she is to continue the use of the medicine for some time.

Should there be any relapse, or if any other cases occur, where I have an opportunity of employing the medicine, I will not fail to communicate to you the result.

I am, Gentlemen,

Your obedient Servant,

PAT. MUDIE.

ARBROATH, SCOTLAND,

*June, 29th,* 1799.

### *Historical Facts relative to the Cure of Consumption.*

In our first volume, p. 290, and foll. and 384, we have announced a new method of treating pulmonary consumption, which was first proposed by Drs. DRAKE and FOWLER; we have also given a concise account of the principles, or the rationale, on which this method was founded, and expressed some doubts, whether the *digitalis* acted as a specific remedy in these cases, or merely produced the effects common to other mild emetics. We

* See our Journal, No. II. p. 184.

We are ſtill inclined to embrace the latter mode of accounting for its action on the arterial ſyſtem, and in juſtice to a ſenſible writer, Francis Fuller, the author of the "*Medicina Gymnaſtica*," printed in 1705, we think it our duty to quote a paſſage from this work which, although not ſtrictly in point, and perhaps involving erroneous principles, evidently points out the method of treating pulmonary conſumption in a manner nearly ſimilar to that lately propoſed by Drs. Drake and Fowler, and ſeconded by Drs. Beddoes, Mossman, and other modern practitioners.

"From theſe conſiderations" ſays Fuller, "of the great ſafety with which we bear vomiting, though it ſeems ſo troubleſome while it laſts, I am convinced that it may be accounted for after the ſame manner as gymnaſtic effects are; beſides that the (temporary or momentary) riſing of the pulſe upon ſtimulating and irritating the fibres of the ſtomach, and the exceſſive preſſure and ſhock of the glands of ſeveral parts with the other phenomena of vomiting, ſhow *that it does partake of the nature of an exerciſe*: and it is a great happineſs for the individual, that the Author of Nature has allotted ſuch ſecondary uſes of the ſtomach, diaphragm, and other parts employed in vomiting, that they ſhould not only ſerve to throw up what is diſagreeable, but *ſtrengthen* the whole economy in that very act. And here it will be allowed, that the irritation of the ventricle does riſe and ſtrengthen much; and I cannot but think I may venture to propoſe it as worthy the conſideration of the beſt judges of theſe matters, whether, when we uſe emetics, we ought to reſt our expectations upon a few momentary efforts, when we ſee nature will bear the carrying on of the ſame meaſures ſo much longer; that is, whether it would not be more expedient, in ſome caſes, to give our ſafe and gentle emetics in leſſer quantities than we do, viz. *ſo as to make a perſon ſick, but not to a degree ſufficient to make him throw up the contents of his ſtomach* and when that quantity of the medicine has paſſed off after that manner, like an alterative, to repeat the ſame doſe, and ſo continue on that ſickneſs *for ſeveral hours*, without raiſing it to that degree, as to force the perſon to vomit above once or twice in all the time. This, I ſay, I think is worth conſideration, and may be *of ſingular advantage in ſome caſes of conſumption*, and in hyſteric caſes, when we do not give vomits to cleanſe the ſtomach only. For, by this means, we can alleviate nature very much, and procure a juſt *diaphoreſis*, when perhaps, by the beſt of cordials, we may only create a colliquation, and after this manner the ſprings will be wound up more gradually; when, if the emetic paſſes off with ſtrong efforts and quick, after the uſual manner, we give nature only a fillip, the effects of which are ſoon over.---I would not be here underſtood as if I would put this in practice; I do not pretend to authority ſufficient for ſuch *innovations*; I only preſume to offer theſe things by way of problem; and one of my ſtature may ſometimes happen to ſtart a *hint* which thoſe who are taller in wiſdom and underſtanding may cultivate and improve to perfection."

The ſame author, in a preceding part of his work aforementioned, treats more at large of *Conſumption*; and though his diagnoſis, prognoſis, and method of cure, are equally imperfect, yet he juſtly cenſures the copious uſe of balſamics and other unctuous internal medicines, which were then much in vogue, while he ſtrongly recommends the uſe of the *colts-foot*, *cynogloſs*, *liquorice*, (without the addition of ſugar) and the *comfreys*.---On a future occaſion, we intend to reſume this important ſubject, which appears to us far from being exhauſted. We ſhall here cite only two other paſſages, from which it will appear that conſumption, about a century ago, was perhaps as common and fatal as it is at preſent; and that equitation, firſt recommended by the illuſtrious Sydenham, was then conſidered as the almoſt only ſpecific in that dreadful diſeaſe.

"I might now proceed farther," ſays Fuller, "to conſider in what degree of this diſtemper riding will be beneficial, whether any thing is to be expected from it, in the ſecond and laſt ſtate of it; but this would be to run run out beyond my defign of brevity; I fhall only take notice that it is no rare thing to meet with confumptions, without any fever, or any reafon to believe an ulcer in the lungs, or perhaps fo much as tubercles, but a continual hectic, and a precipitate wafte of nature by the direful acrimony and ill quality of the ferum, as Dr. BENET, in his "*Theatrum Tabidorum*," obferves, page 109—"Tabidorum languor fine pulmonum aut vifceris cujuflibet corruptelâ tacitâ vi obrepens Anglis infeftiffimus eft, et nifi primis obediverit remediis (quod rariffime evenit) funeftus. In this cafe I cannot but be of opinion, that riding, well managed, would be ferviceable, though undertaken very late, if there is any tolerable meafure of ftrength left to put it in practice.

"I muft here again repeat, that when I fpeak of riding, I underftand the habit of riding, the want of which diftinction has made it ineffectual to many a patient; he that in this diftemper, above all others, rides for his health muft be like a Tartar, in a manner always on horfeback, and then, from a weak condition, he may come to the ftrength of a Tartar. He that would have his life for a prey, muft hunt after it, and when once he finds his enemy give way, muft not leave off, but follow his blow, till he fubdue him beyond the poffibility of a return. He that carries this refolution with him, will, I doubt not, experience the happy effects of the good old direction, *recipe caballum*; he will find that the Englifh pad is the moft noble medium to be made ufe of for a recovery from a diftemper, which we, in this nation, have but too much reafon, by way of eminence, to ftile *Englifh*."

---

### *On the use of the Belladonna, in Cases of Mania.*

In the fixth Number of the "*Journal der Erfindungen*" &c. printed at Gotha, we meet with two cafes communicated by Mr. KÆUFER, Surgeon at Naugardt, in Pomerania, who gives a favourable account of the fpeedy and good effects of the belladona, in that fpecies of mental derangement which is frequently the confequence of epilepfy. He does not, however, mean to infinuate, that this remedy will be of fervice in all fimilar cafes; but he maintains that in the following two cafes it has manifeftly been attended with beneficial effects; on which account he recommends it to the attention of practitioners.

CASE I.—A lady fifty-fix years of age, was firft attacked with epileptic fits, when her menftruation ceafed, at the age of forty-eight; but as the paroxyfms were of fhort duration, and did not return oftener than once in three months, the advice of feveral phyficians whom fhe had confulted, as well as their prefcriptions, were not regularly attended to; after a violent attack of epilepfy, which was followed by all the fymptoms of infanity, Mr. Kæufer was called in: he found her complaining of pain about the pit of the ftomach, and the tongue entirely covered with mucus. Thefe fymptoms induced him to prefcribe emetics, which were from time to time repeated, as they always evacuated much mucus and bile. In the intervals fhe was directed to take a weak folution of tartarum tartarifatum for her common drink; and with a fimilar intention antimonium tartarifatum diffolved in water was added to her beverage. Thefe medicines produced a diarrhœa in which large quantities of black, pitch-like, and very offenfive feces were evacuated. At the fame time, the head was bathed in cold water, and the feet were once a day placed in a decoction of muftard feed; but this method of cure, though continued for three weeks, was not attended with any good effects. The author then had recourfe to the ufe of camphor, which is ftrongly recommended in fuch cafes by MUTZEL and LOCHER: this likewife aggravated the diforder, and the patient became ftill more delirious. After having continued the laft remedy, together with bathing the head and feet in the manner before ftated, Mr. Kæufer refolved upon trying trying the *belladonna*, which has been frequently and succesfully used by Munch (the German Willis), who has lately written, and published several volumes, on the powerful and beneficial effects of this substance, particularly in cases of mania.

Mr. Kæuffer administered *thirteen grains* of the leaves of belladonna, reduced to powder; a dose which produced a profound sleep of thirty-six hours, the patient having had scarcely any sleep during the preceding four weeks. This uninterrupted long sleep, in which she was insensible of being removed from one bed to another, was rather alarming; so that at length the eye-lids were forcibly opened, and while the patient was moderately shaken, a candle was held to the eyes, both pupils of which were much dilated; this expedient, however, had the desired effect of awakening her. During the first half hour after her profound sleep, she spoke rationally, but soon again relapsed into fits of insanity, and uttered incoherent language.

After having taken a second dose, consisting of *ten-grains* of the belladonna, she again fell asleep (it is not stated for what length of time); and after awakening, she found herself in the complete possession of her intellectual powers, and soon enabled to leave her bed.—Six months after this recovery, the patient was again attacked with epileptic fits, accompanied with symptoms of mania; but one dose of ten grains of the belladonna was then sufficient to remove the disease, and she has continued well since that period.

Case II.—Another lady, forty-five years of age, who had likewise been afflicted for several years with epileptic fits, was in a similar manner deprived of her understanding, after a violent paroxysm. Three doses of the belladonna, each containing ten grains, and administered in the space of thirty-six hours, restored her senses; although emetics, purgatives, and cold baths had been previously used, without affording any relief. The sleep of this patient, however, did not continue long, as a profuse perspiration succeeded, which, in the present instance appeared to be critical.

It is with some regret we must, on this occasion, observe to our readers, that medical cases, in general, are so imperfectly stated in foreign Journals, that we seldom meet with accurate pathological descriptions: the illustrious examples of Hippocrates and Sydenham are, alas! every day more neglected; the diagnosis, that *essential* part of medical knowledge, is at present in so despicable a state, that the indications of cure appear to be derived from the shelves of an apothecary's-shop, or the scraps of memorandums and common-place books, rather than from an accurate and methodical inquiry into the nature and seat of diseases, the peculiar constitution of patients, their habits, temperaments, and the particular circumstances connected with the origin of the affection.—Although we are well convinced of the difficulties which present themselves to the practitioner, in forming just and correct indications, when he is called in, as is generally the case, on a sudden emergency, to a patient dangerously circumstanced; although we know, that it is often difficult to institute, in the first and second attempt, such an inquiry into all the particulars of the case, as will lead him to a complete knowledge of the disease; yet we are firmly persuaded, *that the present is exactly the time*, which renders it highly desirable and necessary, that some more effectual and systematic means ought to be adopted, in order to check those deviations from the Hippocratical method of inquiry, and to remedy the baneful effects which they will and must ultimately produce, in the practical department of medicine.

With this intention we have already, in our first Volume, p. 180, inserted a proposal for improving the practice of medicine, by the introduction of Clinical Tables; and as there appears to be but one voice respecting the obvious utility of a *methodical* deſcription of diſeaſes, we ſhall endeavour, at ſome future opportunity, to furniſh the *junior* part of our readers with a ſyſtematic view of the different particulars to which the medical practitioner ought to direct his attention in the examination of patients, as well as in forming a juſt and accurate diagnoſis of diſeaſes. W.

### Cæſarean Operation.

We are ſorry to announce as an article of Medical Intelligence, another unſuccefsful caſe of the Cæſarean Operation, which has been lately performed in the Lying-in Hoſpital at Mancheſter. We had occaſion in our former Numbers to take notice, that the propriety of performing this operation had again become the ſubject of diſcuſſion in that place, and that the controverſy appeared to be conducted with a degree of perſonal aſperity. On this account we were unwilling to enter into the merits of the caſe, leaving the matter where it ſtood before. We felt the more diſpoſed to do this, becauſe there is not any inſtance recorded within our knowledge in this country, in which the Cæſarean Section has not proved fatal to the mother. With a view therefore to the preſervation of the two lives, it has in this country invariably failed; and we have underſtood that where the woman's life was otherwiſe not endangered, it has been generally determined to leſſen the bulk of the child's head *.

The circumſtances which have come to our knowledge reſpecting this unfortunate operation are, that the woman had been delivered in former labours by the crochet, ſeveral times; at the latter end of the laſt month ſhe was again taken in labour, and removed into the Mancheſter Lying-in Hoſpital, where the operation was performed by Mr. Wood, in the preſence of the other medical and chirurgical attendants. The child was extracted alive. During two days ſucceeding to the operation, hopes were entertained of the mother ſurviving, but on the third ſhe died.

It is impoſſible, conſidering the nature of the operation, the violence done to the conſtitution by a large penetrating wound through the parites of the abdomen and the uterus, to attribute her death to any other cauſe; we muſt ſuppoſe that this operation was undertaken with the beſt intentions, but its failure ought to be recorded, that in future it may not be repeated, except under circumſtances where the life of the woman could not be preſerved by any other mode of delivery, unleſs it ſhould be conſidered (which we believe has never been the prevailing ſentiment in England) that the life of the child ought to be preſerved at the expence of the life of the mother.

We ſhould be glad to be favoured with a more particular account of this caſe. As the woman died, the body was moſt probably examined after death, and the dimenſions of the pelvis have been diſtinctly aſcertained, ſo as to determine whether ſhe could have been delivered in any other way.

We ſhould be alſo deſirous of information, whether her deformity had exiſted from her birth, or had been occaſioned by rickets.

### Miſcellaneous Facts and Remarks.

We have the ſatisfaction to announce to our readers, that the experiments lately made on the external uſe of tartarized antimony, by Mr. Hutchinson (of which we have taken notice in a preceding part of this work), are

* Dr. Ozborn's Eſſays on the Practice of Midwifery.

